# Resource Allocation Equity in the China’s Rural Three-Tier Healthcare System

**DOI:** 10.3390/ijerph19116589

**Published:** 2022-05-28

**Authors:** Yibin Ao, Qiqi Feng, Zhongli Zhou, Yunfeng Chen, Tong Wang

**Affiliations:** 1 College of Environment and Civil Engineering, Chengdu University of Technology, Chengdu 610059, China; aoyibin10@mail.cdut.edu.cn (Y.A.); fengqiqi@stu.cdut.edu.cn (Q.F.); 2 College of Management Science, Chengdu University of Technology, Chengdu 610059, China; zzl@cdut.edu.cn; 3 School of Construction Management Technology, Purdue Polytechnic Institute, Purdue University, West Lafayette, IN 47907, USA; chen428@purdue.edu; 4 Faculty of Architecture and the Built Environment, Delft University of Technology, 2628 CD Delft, The Netherlands

**Keywords:** three-tier healthcare system, equity, resource allocation, rural China

## Abstract

The rural three-tier healthcare system is an essential part of the Chinese healthcare service system. To ensure rural residents’ equal access to such healthcare services, it is necessary to examine the current status of the healthcare system in rural China and formulate corresponding improvement suggestions. This study therefore collects the data from the China Health Statistics Yearbook, the China Health Yearbook and the China Statistical Yearbook between the years 2004 and 2021 to calculate the Gini coefficient (G), health resource density index (HRDI) and Theil index (T) first, and then perform the Mann–Kendall test afterwards to evaluate the equity of healthcare resource allocation comprehensively. This series of analysis helps in drawing the following conclusions: (1) county and county-level city medical and health institutions (CMHIs) show a higher development trend in comparison with township hospitals (THs) and village clinics (VCs); (2) VCs have higher institutional fairness, while for beds and personnel, CMHIs and THs are more fairly positioned; (3) more specifically for CMHIs and THs, personnel allocation is more fair than beds and institution allocations; (4) the density of healthcare resources in the eastern and central regions is higher than that in the western part, while the intra-regional distribution of beds and personnel in the west and central regions is better than that in the eastern region; (5) intra-regional differences are more significant than inter-regional differences and the fairness according to population distribution is higher than that of geographical area allocation. The results of this study provide theoretical basis for further optimizing the allocation of healthcare resources and improving the fairness of healthcare resources allocation from a macro perspective.

## 1. Introduction

Globally, health is the eternal theme of human pursuit. In the early 19th century, Charles Winslow, the “father of American Public Health”, proposed that Public Health is a scientific and organized management art that prevents diseases, prolongates life, promotes physical and mental health and improves work efficiency [1]. Currently, social intervention policies, such as the Eight United Nations Millennium Development Goals, are targeting extreme inequalities in healthy well-being, particularly for marginalized people [2]. Fleurbaey recommends that healthcare inequalities should be included in a comprehensive evaluation of social inequalities [3]. The “Healthy China 2030” Planning Outline issued by the State Council proposes that “primary healthcare resources should be rationally distributed according to the permanent residents and service radius so that people can enjoy equal basic healthcare services” [4,5]. The Fifth Plenary Session of the 19th Central Committee of the Communist Party of China put forward the goal of achieving a new level of people’s well-being in the 14th Five-Year Plan, requiring healthcare services to shift from quantity to quality [6].

In China, there is a huge amount of rural population with a long history of poverty which makes rural residents more prone to chronic health problems like cardiovascular and cerebrovascular diseases and metabolic chronic diseases. Furthermore, Chinese rural residents’ cultural backwardness, lack of knowledge and weak awareness of disease prevention and control make the rural chronic diseases more prominent [7]. Furthermore, the level of economic development and healthcare systems varies significantly in different regions. The current government budgeting system and the social medical insurance system are highly dispersed and fragmented. The dual system of excessive and insufficient use of health services still exists [8]. China’s rural areas follow a three-tier healthcare system, which is characterized as a comprehensive county-level medical and health institutions (CMHIs) led, township hospitals (THs) mediated and village clinics (VCs) supported network [9]. The main principles of this system are that residents do not go out of the VCs for minor illnesses, stay in the THs for common illnesses, and pursue help for general serious illnesses in the CMHIs. The predecessor of this three-tier system was introduced and implemented by the rural construction party from 1932 to 1937 [10]. This system was rapidly popularized throughout the country and greatly improved the level of rural healthcare services. By 1978, this healthcare system was highly appraised by the World Health Organization [11].

Since the Reform and Opening-up, the healthcare sector has implemented market-oriented reforms and established a competitive health service market. Problems such as the increasing drug sales, and declining quality of care have led to a near-collapse of this efficient system [12]. In March 2009, the State Council promulgated the ‘Opinions on Deepening the Reform of Health System’ to rebuild a ‘safe, convenient, cheap and effective’ healthcare service system [13], promoting the construction of rural medical facilities and emphasizing the leading role of CMHIs with the support of THs and VCs. In the past decade, the spatial pattern of rural three-tier healthcare service networks have been dramatically reshaped along with the new rural population settlement patterns. Examples are like the centralized distribution of public medical facilities, the expansion of scale and improvement of service quality in the vast rural areas through measures such as ‘removing townships and merging towns’ [14]. China has adopted a variety of initiatives, such as medical alliances (MAs) and hierarchical care systems, aiming to reallocate resources at different levels to meet the needs of residents. According to the medical quality ranking of 195 countries and regions globally by The Lancet, China rose from 60th to 48th in 2015, making it one of the fastest-improving countries [15]. However, there are still many challenges for the three-tier healthcare service system in the rural China. The hierarchical diagnosis and treatment system is still not mature, which makes the time-spending and costs for long-distance treatment enormous. Other challenges are the scarcity of resources, changes in the disease spectrum of residents, aging population, integration of urban and rural development, adjustment of population distribution and the increasing demand of higher level healthcare services [16,17]. The CMHIs-THs-VCs system is imperfect in the sense that patients seek major treatment for minor illnesses and their willingness to go to the grassroots for medical treatment for the first time is very low [18]. This results in the urban medical institutions “a much-visited house” and grassroots medical institutions “few and far between visiting places” [19]. Insufficient and redundant supply of institutions–beds–personnel coexist. Although there are a large amount of rural medical institutions with good accessibility, there is a severe shortage of healthcare resources, obsolete and aging equipment, and low service capacity and quality. These grassroots institutions are faced with the embarrassing situation of “small illnesses cannot be treated well, serious illnesses cannot be cured”. The working conditions of medical personnel are egregious, and the living compensation is not high. The phenomenon of “unable to attract and retain talents” is widespread. At present, there is still a lack of a sustained and long-term coordination mechanism to guide the two-way interaction of healthcare resources in urban and rural areas [20]. 

The development of the eastern–central–western region is unbalanced. The eastern part has a larger population with higher density, a stronger siphon effect [21], and a relatively lower cost of healthcare services. At the same time, the government budgeting process of decentralization means that the affluent eastern provinces have the greater financial capacity to provide funds for healthcare services and provide more financial incentives and career development opportunities to attract high-quality healthcare workers from less developed regions to the eastern areas. However, the agglomeration of high-quality human resources cannot be fully utilized and developed. In particular, the outbreak of COVID-19 at the end of 2019 has brought severe challenges to the public healthcare capacity [22]. The National Health and Health Commission needs to actively engage in COVID-19 prevention and control, activating the epidemic emergency command system, integrating the forces and operating in a flat manner.

Facing these challenges, equity is one of the core elements of the healthcare system to improve, which is of great significance to promoting public health, and ensuring safety [23]. A large number of scholars have conducted research on the fairness of health resource allocation, but most of them have studied a particular field on the macro level: nationally or provincially. Sun [24] and Zhang [12] analyzed the equity of health resources and primary healthcare resources in China, and Li [16] and Zhang [25] evaluated Jiangsu and Shanxi provinces, respectively. Some scholars have also researched CMHIs, THs, and VCs from a micro level. For instance, Li [26] and Tao [27] analyzed CMHIs. Cheng [28] and Audibert [29] measured THs. Zhang [30] and Tan [31] conducted an in-depth investigation on the evolution law of VCs. However, there have been fewer studies on the horizontal comparison of the rural three-tier healthcare system among the three major economic zones [24], and the equalization and salience of different types of healthcare resources.

To evaluate these differences, indicators could be used. Lane systematically reviewed 74 literature and found that operational definitions of equity need to be more precise in addressing different thematic areas before being directly applied to decision-making on the allocation of healthcare resources [32]; Tao [19] found that a single indicator cannot reflect all the allocation gaps, and a comprehensive indicator system should be constructed to evaluate in depth the fairness of healthcare resource distribution as the elements and operation process of the healthcare service system are complex [33]. Furthermore, improper structure and distribution of resource allocation, information asymmetry, poor quality, and inadequate and inappropriate distribution of the healthcare workforce all have an impact on equity. Therefore, it is necessary evaluate the equity based on national conditions from a systematic perspective [20]. 

Therefore, this study aims to use the latest data to systematically evaluate the equity of three types of healthcare resources allocation in three major economic zones for the China’s rural three-tier healthcare system. The research aims to use the analysis to find the causes of deficiencies and explore measures to address them. 

## 2. Data and Methods

### 2.1. Data Sources and Context

In this study, the county-level medical and health institutions (CMHIs) data for 2003 appeared for the first time in the 2004 China Health Statistics Yearbook. Additionally, this is the first time that CMHIs, township hospitals (THs) and village clinics (VCs) data appear simultaneously. Besides the data of healthcare resources, population (household registration) and the geographic areas are also used, and they are derived from the the China Statistical Yearbook, the China Health Statistics Yearbook and the China Health Yearbook from 2004 to 2021. According to the geographical location and economic development level, the provinces, autonomous regions and municipalities in mainland China are divided into three major economic zones: eastern, central and western [34]. 

### 2.2. Indicators and Measurement Methods

Given the requirements of stability, representation, independence, availability and consistency with previous studies, capital and labor are considered important variables in providing healthcare services [16]. The number of institutions and beds represents capital resources, and healthcare personnel represent labor resources [5]. In the Statistical Yearbook, CMHIs include county and county-level city hospitals, maternal and child health hospitals, and specialized disease prevention and control centers. Health personnel include health technicians, rural doctors, health workers, other technicians, managers and workers. VCs have no beds, and to be more in line with the actual situation in the countryside, for VCs’ health personnel resource indication, we have selected the number of rural doctors and healthcare workers.

In order to overcome the limitations of a single research method, such as considering one-sided angle and poor accuracy, this research uses the Gini coefficient (G), the health resource density index (HRDI) and the Theil index (T) to comprehensively evaluate the allocation of healthcare resources. G is used to analyze the difference by population and geography, HRDI is used to combine these two and compare different regions, while T is for measuring the equity changes. In addition, the Mann–Kendall test is for analyzing the trends. We have used Microsoft Excel 2016 software for data entry, calculation of G, T, HRDI and Mann–Kendall non-parametric test values and drawing charts. ArcGIS10.2 software is applied to draw the spatial distribution of HRDI calculation results in 2020.

#### 2.2.1. Gini Coefficients

The Gini coefficient (G) comes from the Lorenz curve, which is an effective tool for assessing the fairness of healthcare resource allocation from both population and geography dimensions [35]. It reflects the ratio of the area between the Lorentz curve and the diagonal to the area below the 45° line. According to international standards [31], *G* less than 0.3 means optimal resource allocation, while 0.3–0.4 represents normal resource allocation and 0.4–0.5 shows that resource allocation is different. *G* between 0.5–0.6 and larger alerts resource allocation [31]. Han [36] made a comparative analysis of eight *G* calculation formulas based on the highest frequency of use, calculation process and practicality. The recommended formula is as follows.

(1) $$G=1-\sum_{i=1}^{n}\left(X_{i}-X_{i-1}\right)\left(Y_{i}+Y_{i-1}\right)$$

where

*G*: the value of the Gini coefficient.
*X_i_*: cumulative percentage of population or geography in the ith district.
*Y_i_*: cumulative percentage of the resources (institutions, beds and health workers) in the ith district.
*n*: total number of districts.

#### 2.2.2. Health Resource Density Index

The health resource density index (HRDI) avoids biases that are solely based on population or geographic areas. Formula (2) is used to calculate the HRDI.

(2) $$HRDI=\frac{HR_{i}}{\sqrt{A_{i}P_{i}}}$$

where:

*HRDI*: the value of the health resource density index.
*HR_i_*: health resource quantity of the ith region.
*A_i_*: geography of the ith region.
*P_i_*: population of the ith region.

#### 2.2.3. Theil Index

The advantage of the Theil index (T) is that it can analyze the root causes of inequity and measure the contribution of intra-regional and inter-regional differences to the overall inequality [31]. Equation (3) is used to calculate the $T_{\mathrm{total}}$. The $T_{\mathrm{total}}$ can be decomposed into the $T_{\mathrm{intra}}$ and $T_{\mathrm{inter}}$ [37]. Equation (4) and Equation (5) are used to calculate the inter-region and intra-region *T*, respectively. The value range of the *T* is [0, 1]; the closer the result is to 0, the higher the fairness. The contribution rate of intra- and inter-region can be calculated by dividing $T_{\mathrm{total}}$, as $T_{\mathrm{intra}}$/$T_{\mathrm{total}}$ and $T_{\mathrm{inter}}$/$T_{\mathrm{total}}$.

(3) $$T_{\mathrm{total}}=\sum_{i=1}^{n}\frac{P_{i}}{P}\log\left(\frac{P_{i}/P}{E_{i}/E}\right)=T_{\mathrm{inter}}+T_{\mathrm{intra}}$$

$$T_{\mathrm{total}}=T_{\mathrm{inter}}+T_{\mathrm{intra}}$$

(4) $$T_{\mathrm{inter}}=\sum_{g=1}^{k}\frac{P_{g}}{P}\log\left(\frac{P_{g}/P}{E_{g}/E}\right)$$

(5) $$T_{\mathrm{intra}}=\sum_{g=1}^{k}\frac{P_{i}}{P}\log\left(\frac{P_{i}/P_{g}}{E_{i}/E_{g}}\right)$$

where

$T_{\mathrm{total}}$, $T_{\mathrm{inter}}$, $T_{\mathrm{intra}}$: represent the total, inter- and intra-Theil index, respectively.
$P_{i}$, $P_{g}$, P: represent the population or geographic area of each province, three major economic regions, and the whole country, respectively.
$E_{i}$, $E_{g}$, E: represent the medical and health resources of each province, the three major economic zones, and the whole country, respectively.

#### 2.2.4. Mann–Kendall Test

Mann–Kendall nonparametric rank test is very useful in data trend detection, and it is often used to determine whether there is a climate mutation in the climate sequence. If there is, the time of the mutation can be determined. Neel Kamal [38] and Khaled H. Hamed [39] described the method in detail in their study. In this study, the test is used to detect the trend of *G*, HRDI and *T*, which can provide reference for the next policy formulation. The test statistic *S* obeys normal distribution, and *Z* (*c*) statistic (Formula (8)) is calculated by *S* statistic (Formula (6)) and variance ${\mathrm{Var}}_{\left(s\right)}$ (Formula (7)). A positive *Z* value indicates an increasing trend. The statistical test criteria are set to α = 0.01, 0.05, 0.1, respectively [40].

(6) $$S=\sum_{i=1}^{n-1}\sum_{j=i+1}^{n}sgn\left(X_{i}-X_{j}\right)\;\;\;\;\;\;\;\;\;\;\;\;\;\;\;j>i\geq 1$$

(7) $${\mathrm{Var}}_{\left(s\right)}=\frac{1}{18}\left[n\left(n-1\right)\left(2n+5\right)-\sum_{t}f\left(t\right)\left(f\left(t\right)-1\right)\left(2f\left(t\right)+5\right)\right]$$

(8) $$Z\left(c\right)=\{\begin{array}{c} \frac{S-1}{\sqrt{{\mathrm{Var}}_{\left(s\right)}}}\;,\;\;S>0\\ 0,\;\;\;\;\;S=0\\ \frac{S+1}{\sqrt{{\mathrm{Var}}_{\left(s\right)}}}\;,\;\;S<0 \end{array}$$

## 3. Results

This section firstly presents the past trend regarding healthcare resources allocation in the rural China (Section 3.1), followed by a detailed analysis of equity using Gini coefficient (G), the health resource density index (HRDI) and Theil Index (T) and Mann–Kendall tests (Section 3.2).

### 3.1. Status Quo

**Figure 1:**
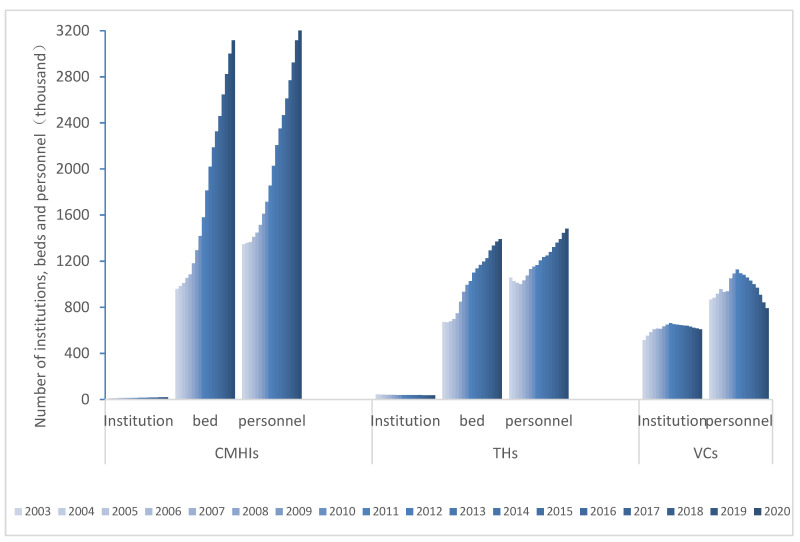
The past trend of resource allocation in China’s rural three-tier healthcare system from 2003 to 2020 (CMHIs: county and county-level city medical and health institutions; THs: township hospitals; VCs: village clinics).

From 2003 to 2020, tremendous changes have taken places in rural medical care in China. As shown in Figure 1, from the perspective of time series, the county and county-level city medical and health institutions’ (CMHIs) numbers of institutions, beds, and personnel have shown an increasing trend, with an average growth rate of 3.15%, 7.23%, and 5.35%, respectively. Township hospitals (THs) actively respond to the requirements of village-dismiss and town-combination. Although the number of institutions has decreased 1.24%, numbers of beds and personnel have increased, with an average growth rate of 4.42% and 2.02%, respectively. The numbers of institutions and personnel in the village clinics (VCs)s show an increasing trend from 2003 to 2011, and a decreasing trend from 2012 to 2020. The average growth rates are 1.02% and −0.43%, respectively. From the cross-sectional data, the VCs institution numbers were 45 times and 11.63 times the numbers of CMHIs and THs in 2003, and the personnel were 0.64 times and 0.82 times, respectively. Compared to 2003, by 2020, the institution changes were 31.52 times and 17.02 times, and the personnel decreased sharply to 0.24 times and 0.53 times. The number of beds in CMHIs divided by VCs has dropped sharply from 0.7 times in 2003 to 0.45 times in 2020.

### 3.2. Equity in the China’s Rural Three-Tier Healthcare Service System

#### 3.2.1. Gini Coefficients Results

**Figure 2:**
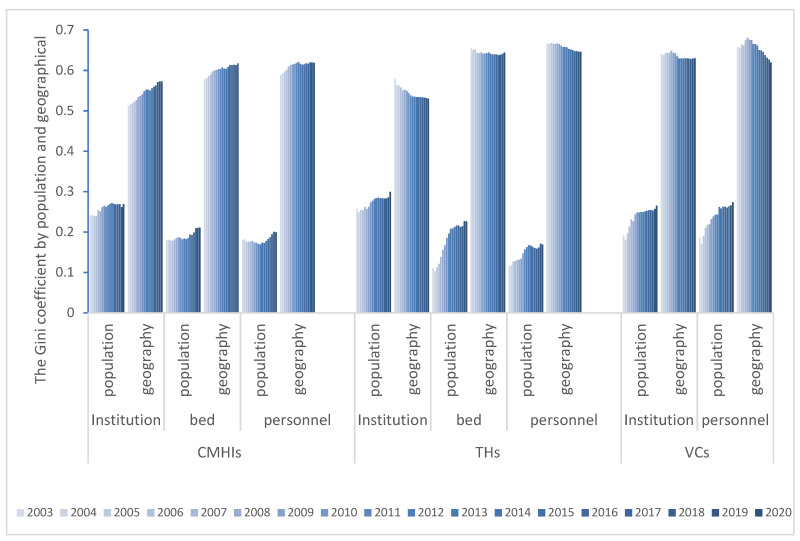
The Gini coefficient for healthcare resources by population and geographic areas in China from 2003 to 2020 (CMHIs: county and county-level city medical and health institutions; THs: township hospitals; VCs: village clinics).

The Gini coefficient by population and geographical area from 2003 to 2020 is shown in Figure 2. By population, the G of CMHIs, THs and VCs are 0.169–0.271, 0.103–0.299, and 0.17–0.273, respectively, showing a good fairness. However, it is worth noting that there is an increasing trend that the fairness is decreasing. Among them, the mean values of institutions, beds, and personnel numbers in CMHIs are 0.259, 0.189, and 0.180, and the total growth rates are 11.4%, 17.19%, 11.11%, respectively. The average values of them in the THs are 0.273, 0.179, 0.148, and the growth rates are 16.13%, 105.44% and 45.85%, respectively, with the most serious trend of inequity in beds. The average value of VCs is 0.237, and the growth rates are 38.85% and 47.21%, respectively In recent years, the G of CMHIs and THs show the distribution of institutions are the most uneven. At the same time, the G of the VCs indicates that the inequity of personnel is the utmost. The partiality of institutions and beds manifest the value of THs is the largest, while that of VCs is the smallest, and the injustice order of personnel is just the opposite.

The G of CMHIs according to the geographical area are 0.512–0.621, THs are 0.529–0.668, and VCs are 0.619–0.681, showing a state of alertness and danger with poor fairness. From the time series, in addition to the increasing G of CMHIs and the increasing inequality, the values of THs and VCs are decreasing, which ameliorates the inequality. Among them, the CMHIs’ average figures of the three types of health resources are 0.545, 0.602 and 0.612, and the total growth rates are 11.87%, 7.05% and 5.15%, respectively. The average values of THs are 0.544, 0.643 and 0.657, and the growth rates are −8.59%, −1.62% and −3.02%, respectively. The average values of VCs are 0.636 and 0.655, and the growth rates are −1.63% and −5.75%, respectively. Moreover, in recent years, the distribution by population is just the opposite. The partiality of CMHIs and THs demonstrates that the distribution of personnel is the worst, while the numbers of VCs institutions exceeds personnel. The inequity of institutions and personnel in VCs performs worse, and for the beds injustice order, THs are more unfair.

#### 3.2.2. Health Resource Density Index Results

**Figure 3:**
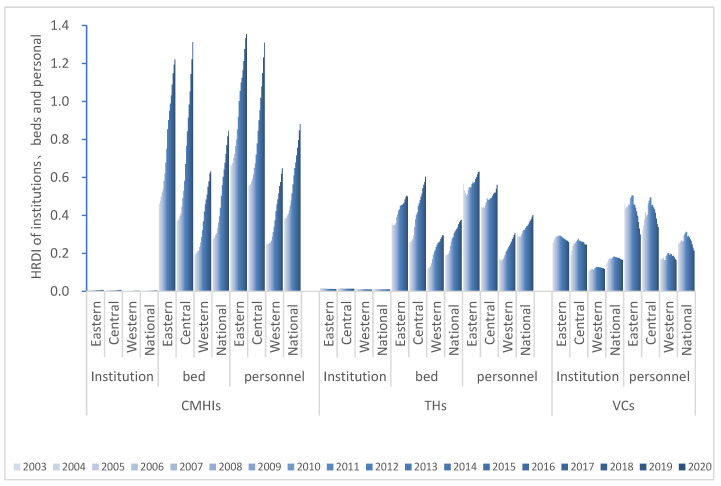
The health resource density index (HRDI) for healthcare resources by Eastern, Central, Western, National in China from 2003 to 2020 (CMHIs: county and county-level city medical and health institutions; THs: township hospitals; VCs: village clinics).

As shown in Figure 3, the HRDI of the eastern and central regions is greater than that of the whole country, and the western part is smaller than that of the national region. Moreover, the central area has the most expeditious growth rate and the sharpest development curve. Eastern, central, western and national data all show that the fairness of institutions is the worst. Additionally, the CMHIs have the most health resources.

**Figure 4:**
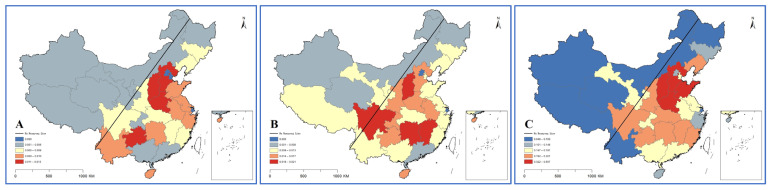
HRDI distribution map of institutions in 2020 ((**A**) for county and county-level city medical and health institutions; (**B**) for township hospitals; and (**C**) for village clinics).

**Figure 5:**
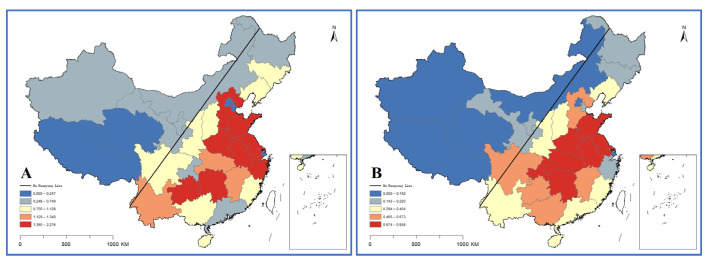
HRDI distribution map of beds in 2020 ((**A**) for county and county-level city medical and health institutions; and (**B**) for township hospitals).

**Figure 6:**
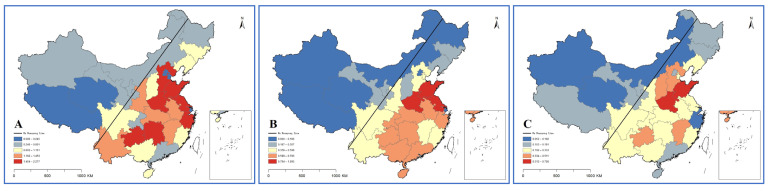
HRDI distribution map of personnel in 2020 ((**A**) for county and county-level city medical and health institutions; (**B**) for township hospitals; and (**C**) for village clinics).

Combining HRDI with the data map vividly shows the differences among provinces, autonomous regions and municipalities in 2020 (Figure 4, Figure 5 and Figure 6). Through the fusion with ArcGIS, it can be seen that there is a growth trend from northwest to southeast, which is in line with the spatial distribution law of the “ Hu Huanyong Line ” [41]. The median area and high-value area are concentrated in the east of the Hu Huanyong Line, and the low-value area is mainly distributed in the west of the line. 

#### 3.2.3. Theil Index

The Theil index has the analogous trend as the G by population. The $T_{\mathrm{total}},\;T_{\mathrm{inter}}$ and $T_{\mathrm{intra}}$ of CMHIs and THs shows that the distribution of institutions is the most unbalanced. The T values of VCs indicate that the fairness of personnel is the lowest. The $T_{\mathrm{total}},\;T_{\mathrm{inter}}$ and $T_{\mathrm{intra}}$ of the institution are showing that this resource type for CMHIs exceeds the levels in VCs. The $T_{\mathrm{total}}$ and $T_{\mathrm{intra}}$ of the bed all display that such resource allocation in CMHIs are more unfair than in THs, while for $T_{\mathrm{inter}}$ the situation is the opposite. The $T_{\mathrm{total}},\;T_{\mathrm{inter}},\;T_{\mathrm{intra}}$ of the personnel are the highest values in the VCs, and the inequity is the greatest.

**Figure 7:**
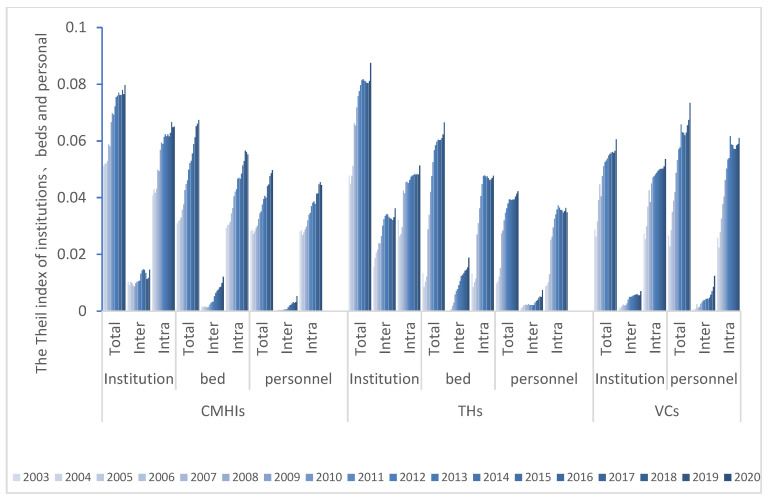
Changes in Theil index of health resource allocation from 2003 to 2020 (CMHIs: county and county-level city medical and health institutions; THs: township hospitals; VCs: village clinics).

**Figure 8:**
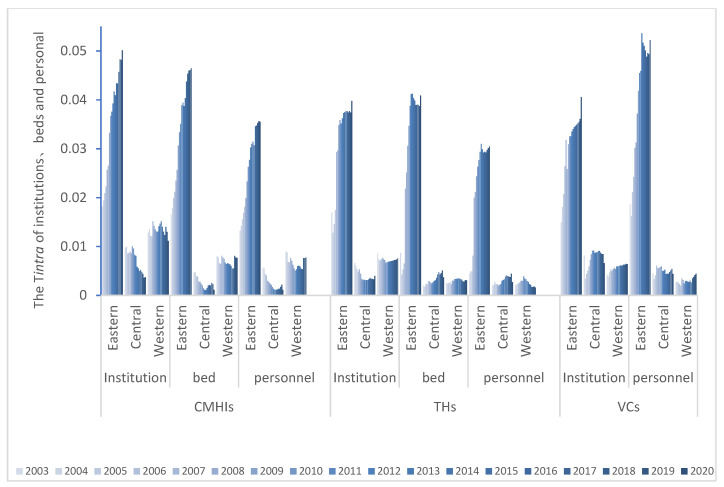
Changes in $T_{\mathrm{intra}}$ of health resources allocation in the eastern, central and western regions from 2003 to 2020 (CMHIs: county and county-level city medical and health institutions; THs: township hospitals; VCs: village clinics).

Further analysis shows that the trends of $T_{\mathrm{inter}},\;T_{\mathrm{intra}}$ and $T_{\mathrm{total}}$ are consistent (Figure 7). The average intra-group contribution rates are much larger than the intra-group to the overall differences, but both have a decreasing trend, meaning the inequity has been enlarged (Table 1). Continuing to decompose the differences within the region, the T of the eastern part is generally more significant than that of the central and western areas. The internal difference is the largest, and the inequity is still expanding. In contrast, the distribution of healthcare resources in the west and central regions is relatively balanced, and the equity is remedied (Figure 8). 

#### 3.2.4. Mann–Kendall Test

The Mann–Kendall nonparametric test is performed on the G distributed by population and geographic areas, and the trend test results are shown in Table 2. It can be seen that when the population is used as the measurement calibre, the G all show an upward trend. Among them, except for the CMHIs’ personnel, which is not statistically significant (*p* > 0.1), the increasing trend of other indicators are pronounced (*p* < 0.01). When the geographical area is used as the measurement calibre, except for the CMHIs, the G of the THs and VCs show a downward trend, and the difference is statistically significant (*p* < 0.01).

Mann–Kendall nonparametric tests are performed on $T_{\mathrm{total}},\;T_{\mathrm{inter}},\;T_{\mathrm{intra}}$, respectively, and the trend test results are shown in Table 3. It can be found that all T have an upward trend and are statistically significant (*p* < 0.01).

The Mann–Kendall test results of HRDI in eastern, central, western and national regions are shown in Table 4. CMHIs and THs have significant statistical significance (*p* < 0.01), except for the THs, which are decreasing, the other indicators show an increasing trend. At the same time, the institutions and personnel in the eastern part of the VCs offer a downward trend, and the institution figures in western regions have an increasing proneness (*p* < 0.1).

## 4. Discussion

After a long period of reform and development, China’s rural three-tier healthcare service system has rapidly changed. This research finds that the county and county-level city medical and health institutions (CMHIs) are developing the fastest. Although the number of institutions in township hospitals (THs) are decreasing, the numbers of beds and personnel are rising. The development prospect of the village clinics (VC) is not optimistic, and there has been a decline in healthcare resources and continued widening gaps due to the highly dispersed healthcare service system, the lack of interaction between market mechanisms, and the merger of administrative villages.

The results of Gini coefficients (G) show that the distribution of medical and health resources is generally fair, and the fairness of distribution by population is higher than that by geographical areas; this is consistent with the findings of Zhang et al. [30]. When the population is used as the standard, the contribution of institutions to equity is the smallest, and the number of beds or personnel mainly causes the reduction of the G. When using the geographic area as the standard, institutions make the most outstanding contribution to fairness, and unfair staffing is the main reason for the high G, which is consistent with the findings of Xu [11]. Most of the documents issued by the Chinese Government aiming to optimize the allocation of healthcare resources are based on population, and the focus on the geographical portion of resources is relatively lacking. At the same time, the injustice of CMHIs and THs are reflected by the institution numbers according to population distribution, and personnel is more unfairly allocated than beds and institutions according to geographical distribution. In the VCs, the values show just the opposite. An effective referral system will thus enable medical institutions to “perform their respective responsibilities and do their own things”, thus a hierarchical diagnosis and treatment system with “clear top and bottom, smooth circulation” is needed [16]. CMHIs, THs and VCs institutions are encouraged to strengthen vertical business cooperation and classify diseases according to the severity and urgency of diseases. Institutions at all levels carry out two-way referral work according to their clear functional orientation, so that medical institutions at different levels can deal with different types and stages of diseases according to the function of institutions, and guide rural residents to make reasonable use of medical and health resources [42]. Finally a medical and healthcare service network system should be formed with suitable service radius, convenient transportation, moderate quantity and complete coverage, so as to alleviate the practical problems such as “inadequate leading role”, “not working hub”, and “the weak bottom of the network”.

The results of the health resource density index (HRDI) are generally consistent with those of G (by population). The HRDI of the eastern and central regions is greater than that of the whole country, and the western part is less than the whole country, Sun [24,43] and Li also reached same conclusions. The development trend of VCs is that institutions are faster than personnel, and VCs have the largest number and the widest distribution. CMHIs on the other hand have the most health resources. The Hu Huanyong Line shows that the level of economic development has a certain impact on the regional differences in resource allocation of medical institutions [43]. 

China has a vast territory, complex and diverse topography, and uneven population distribution. Therefore, when carrying out healthcare planning in the future, we should comprehensively consider factors such as service population, geographical heterogeneity, spatial accessibility, economic development level, medical and healthcare market outline, and planning for equality according to local conditions. Promoting public health planning with geospatial analyses also can help in defining a scientific geographic unit for the healthcare market [17]. The government should take several measures such as promoting the equalization by coordinating development of rudimentary medical services in an all-around way with increasing support for underdeveloped and remote areas in the west to narrow the gap. It is also needed to maintain and coordinate the development among the east, middle and west [17,32].

Theil index (T) indicates that the distribution of institutions is the most unbalanced, followed by beds. Zhang [44] argues that the redistribution of beds has dramatically improved the fairness of space access. The order of the VCs is precisely the opposite, and the brain drain is the most serious. The decomposed T and the overall T change in the same trend, and the gap is mainly due to intra-regional differences, which is much greater than the contribution rate of inter-regional to the overall differences [44,45]. It indicates that the differences in healthcare resource allocation among regions with different levels of economic development are obvious. However, they all have a decreasing trend, which has enlarged the unfairness. At the same time, it implies that the main reason for the unfair allocation of healthcare resources is from the unfair distribution within the region and beyond to inter-regional distribution, which is the same as Zhang’s [11] research results [12]. Continuing to decompose the intra-regional differences, the T of the eastern part is generally more prominent than the central and western regions. Rapid industrialization and urbanization have made the internal differences in the eastern region the largest, while the distribution system in the west and central areas is better. Inclined policies and assistance have improved the fairness of the west and central regions. In addition to the large contribution of personnel in the eastern part of the VCs, the contribution of personnel in the eastern, central and western CMHIs and THs, and the west and central parts of the VCs is relatively small, and the distribution system is better. The allocation of resources should adopt the strategy of “total control and internal structure optimization” [46] to improve the marginal utility of health resources [47]. On the one hand, we should insist on strengthening “hardware” construction. The Matthew effect in remote areas is serious, and the government’s macro-control efforts are low, and so is the investment there in healthcare. On the other hand, resources are limited and operating costs are high. Therefore, the small population creates more complexity and fragmentation of the healthcare system in rural China. The government should adhere to Pareto’s improvement by providing additional subsidies and other preferential policies and pay attention to the vertical integration and horizontal transfer of healthcare resources [4]. Furthermore, the use of Internet and Smart Medical technology could be considered to promote information empowerment and accelerate the upgrade of advanced medical knowledge and technology [46]. Furthermore, it is necessary to promote the improvement of “software”, to actively implement the hierarchical diagnosis and treatment policy and innovate the medical service mode by relying on the construction of medical alliance, medical community, and the remote diagnosis and treatment system [4].

According to the significant degree of *p* value in Table 2, Table 3 and Table 4 and the trend of increase or decrease, we can find that *G*, HRDI and *T* are consistent with the results of the Mann–Kendall test, and most of them are statistically significant. When the G is distributed according to population, it tends to increase, and when it is distributed according to the geographical area, the values of THs and VCs decrease, except for those of CMHIs. HRDI shows that in addition to the decreasing trend of THs and VCs’ institutions and personnel in the east, their indexes generally show an increasing trend. T is a rising trend, the future will also tend to be unfair, and the development trend is not optimistic, so special attention should be paid to it. Therefore, it is recommended that the decisive role of the market in the allocation of medical resources should be strengthened, and more attention to the guiding function of the government in the allocation of medical resources should be given [48]. In the next stage, the focus of China’s medical system reform will shift from “extending the coverage” to solving the imbalance and insufficient contradiction in the field of medical security [49]. Based on the law of land space development and construction in the new era [42], the dual structure of urban and rural areas will be changed gradually to integrate the medical insurance system and guide the health resources to be tilted towards rural areas. The optimal layout of medical facilities should be based on more accurate prediction of the rural population and urban system, and the areas with a large population and scattered residence can be added as appropriate. In contrast, the areas with a small population and close distance should be combined to allocate healthcare resources more efficiently and effectively.

Although this research systematically analyzes the status quo and causes of fair allocation of healthcare resources in China through various methods, there are still some limitations. Firstly, quantifying the three types of health resources is relatively rough. For example, institutions are not subdivided into the technical level, registration type, sponsor, etc., and personnel are not detailed by gender or age. Differences between doctors and nurses, and beds are not analyzed according to the department, and the target population was the resident population, and did not consider the difference of floating population on the authenticity of the impact. Secondly, the data used lags behind the most representative statistical data at present, reducing the timeliness of decision-making. Finally, some indicators such as government health expenditures, financial subsidies and other data are unavailable, so they cannot be further evaluated from a financial perspective, and the adjustment of statistical calibre may affect the accuracy of the evaluation. Based on this, follow-up research can formulate a more detailed study on the current weak links of equity in China according to the actual health status and healthcare service needs of different populations in different regions.

## 5. Conclusions

China urgently needs to establish an effective rural healthcare management system and operation mechanism. At present, a large number of scholars have studied the fairness of health resource allocation, but many studies are aimed at the changes after the new medical reform in 2009, and there is little comprehensive long-term dynamic change research. Furthermore, most of the existing research studies a specific level of institutions from the macro level, while there are few studies evaluating the fairness of resource allocation at different levels [4,48]. In addition, the existing research methods are single and the results lack accuracy. Comprehensive indicators can effectively improve the accuracy, so as to evaluate the fairness of health resource allocation more comprehensively and deeply [50,51,52].

Therefore, this study considers the distribution of different types of medical and health resources (institutions, beds, personnel) in different levels of medical and health institutions, and the differences in resource changes between regions and regions (eastern, central, western, and national), and compares the overall distribution in different spaces. Since the county and county-level city medical and health institutions (CMHIs) data of 2003 appeared for the first time in the China Health Statistical Yearbook in 2004, the time span selected in this study was from 2003 to 2020, in order to fully reflect the trend of resource allocation. The fairness difference of health resource allocation is analyzed in the 18-year time dimension with several normally applied indicators.

This study thus comprehensively evaluates the equity of resource allocation in rural China’s three-tier healthcare service system. The results of the study could help adjust the stock of medical and healthcare resources scientifically and make the healthcare service system more rational, with clear division of labor and more targeted functions. Some recommendations are formulated based on the systematic analysis. First, attention should be given to the leading role of CMHIs and district medical and health institutions, and there should be implementation of vertical and horizontal linkages with large and small, and resource sharing. Second, we recommend running a health center in each township hospital (TH), as THs play a pivotal role in health services. Third, we suggest taking various forms of ‘supporting the construction of village vlinics (VCs)’ to make it conduct the fundamental role of service outlets. Four, prescribe the proper remedy to avoid treating the symptoms but not the root cause, and build China’s rural three-tier healthcare network into a healthcare service system with sound institutions, clear responsibilities, coordination and cooperation, and effective operation.

## Figures and Tables

**Table 1:** Proportion of intra-group contribution differences of institutions, beds and personnel, based on China’s rural three-tier healthcare system (CMHIs: county and county-level city medical and health institutions; THs: township hospitals; VCs: village clinics).

Year	CMHIs (%)			THs (%)			VCs (%)	
	Institution	Bed	Personnel	Institution	Bed	Personnel	Institution	Personnel
2003	79.80	94.86	98.97	67.38	99.52	89.51	95.21	97.40
2004	82.51	95.71	99.27	58.51	99.87	86.92	96.55	98.56
2005	80.05	94.86	98.32	56.83	98.51	83.51	94.50	97.43
2006	81.37	95.26	98.61	57.81	94.51	85.56	94.11	93.21
2007	84.78	96.26	98.74	63.95	93.68	91.81	95.56	96.94
2008	85.11	96.30	99.09	63.51	91.29	92.30	94.96	96.31
2009	85.27	94.90	98.54	63.25	86.22	92.18	94.27	94.70
2010	85.19	93.90	98.51	60.23	85.20	93.85	92.18	94.27
2011	84.99	93.15	98.37	58.23	85.35	94.03	90.59	93.56
2012	85.16	93.43	98.39	57.99	83.92	94.39	90.73	93.46
2013	82.53	89.99	96.62	58.16	81.80	94.57	90.54	93.71
2014	81.18	88.00	95.24	58.32	79.37	92.58	90.14	93.17
2015	81.00	87.21	94.54	59.07	78.78	91.18	89.84	93.13
2016	80.94	87.23	93.98	59.67	77.79	90.20	89.77	92.36
2017	82.43	86.39	92.97	59.69	76.46	88.28	89.34	90.83
2018	85.58	86.89	93.97	60.16	76.09	87.33	90.00	89.27
2019	84.75	84.97	93.48	59.25	75.31	87.94	90.20	87.36
2020	81.65	82.01	89.38	58.64	71.72	82.49	88.41	83.11
mean	83.02	91.18	96.50	60.04	85.30	89.93	92.05	93.27

**Table 2:** Mann–Kendall test results of the Gini coefficient (CMHIs: county and county-level city medical and health institutions; THs: township hospitals; VCs: village clinics).

Measurement Calibre	CMHIs			THs			VCs	
	Institution	Bed	Personnel	Institution	Bed	Personnel	Institution	Personnel
Population	3.409 *** (+)	4.242 *** (+)	1.364 (+)	4.318 *** (+)	5.227 *** (+)	4.470 *** (+)	5.379 *** (+)	5.303 *** (+)
Geography	5.606 *** (+)	5.303 *** (+)	4.091 *** (+)	−5.606 *** (−)	−3.409 *** (−)	−5.151 *** (−)	−3.106 *** (−)	−3.185 *** (−)

*** *p* < 0.01, (+) Represents an increasing trend and (−) represents a decreasing trend.

**Table 3:** Mann–Kendall test results of the Theil index (CMHIs: county and county-level city medical and health institutions; THs: township hospitals; VCs: village clinics).

Measurement Calibre	CMHIs			THs			VCs	
	Institution	Bed	Personnel	Institution	Bed	Personnel	Institution	Personnel
Total	5.303 *** (+)	5.757 *** (+)	5.379 *** (+)	4.318 *** (+)	5.379 *** (+)	5.379 *** (+)	5.454 *** (+)	5.000 *** (+)
Inter	3.409 *** (+)	5.000 *** (+)	5.076 *** (+)	3.939 *** (+)	5.682 *** (+)	4.167 *** (+)	4.924 *** (+)	5.530 *** (+)
Intra	5.076 *** (+)	5.454 *** (+)	5.151 *** (+)	5.076 *** (+)	3.788 *** (+)	3.712 *** (+)	5.606 *** (+)	4.545 *** (+)

*** *p* < 0.01, (+) Represents an increasing trend.

**Table 4:** Mann–Kendall test results of HRDI (CMHIs: county and county-level city medical and health institutions; THs: township hospitals; VCs: village clinics).

Measurement Calibre	CMHIs			THs			VCs	
	Institution	Bed	Personnel	Institution	Bed	Personnel	Institution	Personnel
Eastern	5.606 *** (+)	5.757 *** (+)	5.757 *** (+)	−5.757 *** (−)	5.151 *** (+)	4.470 *** (+)	−1.742 * (−)	−2.197 ** (−)
Central	5.379 *** (+)	5.757 *** (+)	5.757 *** (+)	−5.151 *** (−)	5.757 *** (+)	5.151 *** (+)	0.530 (−)	0.076 (+)
Western	5.151 *** (+)	5.757 *** (+)	5.682 *** (+)	−5.530 *** (−)	5.757 *** (+)	5.303 *** (+)	1.894 * (+)	0.455 (+)
National	5.530 *** (+)	5.757 *** (+)	5.757 *** (+)	−5.757 *** (−)	5.682 *** (+)	5.227 *** (+)	0.379 (+)	−0.379 (−)

*** *p* < 0.01, ** *p* < 0.05, * *p* < 0.1, (+) Represents an increasing trend and (−) represents a decreasing trend.

## Data Availability

The data are available from the first author upon reasonable request.
